# Age and Gender Differences in Home Injury Prevention Awareness and Behaviors Among Community-Dwelling Older Adults

**DOI:** 10.3390/healthcare14010049

**Published:** 2025-12-24

**Authors:** Ok-Hee Cho, Hyekyung Kim

**Affiliations:** 1 Department of Nursing, College of Nursing and Health, Kongju National University, Gongju 32588, Republic of Korea; ohcho@kongju.ac.kr; 2 Department of Nursing, Catholic Kwandong University, Gangneung 25601, Republic of Korea

**Keywords:** older adults, injury, prevention, home environment

## Abstract

**Highlights:**

**What are the main findings?**

- The rates of first-aid awareness, possession of emergency kits, and participation in regular health check-ups declined with increasing age.
- Intention to participate in preventive education and engagement in preventive behaviors were higher among women than men.

**What are the implications of the main findings?**

- Approaches that account for age- and gender-specific differences in both awareness and behavior are needed.
- Tailored educational and policy initiatives may enhance safety among older adults and reduce the physical, and social burden of household injuries.

**Abstract:**

Background/Objectives: This study aimed to examine awareness and behaviors related to injury prevention in the home among community-dwelling older adults according to age and gender. Methods: In this cross-sectional descriptive study, 299 adults aged ≥65 years who visited 10 senior welfare centers in Korea were included. Data were collected through structured face-to-face interviews using a questionnaire assessing general characteristics, awareness, and behaviors related to home injury prevention. Descriptive statistics, the chi-squared test, independent t-test, and one-way analysis of variance were used for data analysis. Results: Levels of interest in home injuries and awareness of first aid differed significantly by age and gender. The possession of a home emergency kit and participation in regular health check-ups varied by age, while the intention to participate in injury-prevention education differed by gender. Overall, female participants demonstrated higher levels of injury-prevention behavior than male participants. Conclusions: Older adults showed lower awareness and practice of specific home injury–prevention strategies, while women exhibited greater awareness and preventive behaviors than men. These findings suggest that tailored home injury–prevention interventions that consider age and gender characteristics may yield more effective and positive outcomes.

## 1. Introduction

Globally, the proportion of people aged ≥65 years is projected to increase from ~10% in 2022 to 16% by 2050 [1]. Korea is one of the countries experiencing the most rapid demographic aging; as of 2024, older adults account for 19.2% of the total population, and this figure is expected to reach 40% by 2050 [2]. The accelerated growth of the older population, combined with age-related physiological decline and chronic conditions such as stroke and dementia, substantially heightens the risk of unintentional injuries [3,4]. Older adults are particularly vulnerable to serious harm even from minor incidents, making them a high-risk group for home and community injuries [5]. Although the home is typically regarded as a safe environment, it can be one of the most common sites of injury in older adults. Studies in Korea [6] and India [7] have reported that more than half of all accidents occur within the home. Falls are the leading cause of household injuries, but other events, such as cuts from sharp objects, burns, and poisonings, also occur frequently [7]. Feeling unsafe at home can increase stress, lower self-efficacy, and diminish perceived control, ultimately reducing health-related quality of life among older adults [8].

For injury-prevention programs targeting older adults to be effective, it is essential that individuals first recognize injuries as preventable occurrences [9]. Personal safety behaviors and environmental modifications are effective strategies for preventing unintentional home injuries [10]. However, these preventive actions are likely to occur only when older adults themselves are aware of the risks and perceive injury prevention as personally relevant [9]. Many older adults have limited exposure to formal safety education and often fail to perceive potential hazards in the home [11]. They tend to modify their behavior only when they recognize a risk as directly relevant to them [12]. Injury incidence increases with age [13,14], and injury-related mortality and hospitalization rates are highest among adults aged >75 years [15]. As advancing age is frequently accompanied by deteriorating health, prolonged time spent at home and marked declines in mobility and activities of daily living, older adults’ perception of injury risk may vary depending on these functional and environmental changes [16]. Therefore, identifying age-related differences in awareness and experience of home injuries is crucial for developing appropriate interventions. 

Gender differences also play a significant role in injury occurrence and prevention awareness. In Korea, older women accounted for 63% of all hospital discharges due to injury, 1.3 times higher than the number of men [17]. Similarly, in Israel, 70% of home injuries occurred among older women compared with 30% among older men [18]. This discrepancy reflects differences in daily roles and exposure patterns: women are more likely to sustain burns during cooking, while men are more prone to cuts and lacerations during home repairs or do-it-yourself activities [18]. Such gender-based variations in the frequency and type of injuries may influence awareness and engagement in preventive behaviors.

Despite its prevalence, home injury among older adults has received limited attention from policymakers and researchers compared with injuries occurring in public or occupational settings. A detailed understanding of subgroup characteristics such as age and gender is necessary to determine which aspects of home safety should be prioritized [19]. Most prior research has focused on describing the incidence and risk factors of home injuries among older adults [3,5,18,20]; however, little is known about their awareness of injury prevention and their actual preventive behaviors. Against this backdrop, this study aimed to investigate awareness and practices related to home injury prevention among community-dwelling older adults, according to age and gender, to provide foundational data for developing effective, tailored prevention programs.

## 2. Materials and Methods

### 2.1. Participants and Data Collection 

This study employed a cross-sectional design using structured questionnaires. Participants were older adults who visited 10 senior welfare centers in Korea. Participants were recruited using a convenience sampling approach. Center staff were not involved in identifying, selecting, or inviting participants. Among those who voluntarily agreed to participate, individuals who met the inclusion criteria were surveyed. The inclusion criteria were as follows: (1) aged ≥65 years, (2) residing in their own homes, and (3) able to understand and independently respond to the questionnaire. Exclusion criteria included a diagnosis of psychiatric disorder or dementia, current use of psychiatric medication, and inability to communicate effectively. The required sample size was calculated using the G*Power program version 3.1. With a significance level of 0.05, statistical power of 0.95, and an effect size of 0.25, a minimum of 252 participants was required. A total of 310 questionnaires were distributed, and 300 were returned (response rate = 99.0%). After excluding one incomplete response, data from 299 participants were included in the final analysis.

### 2.2. Instruments

#### 2.2.1. General Characteristics

Eight items were included to assess participants’ general characteristics: age, gender, educational level, marital status, employment status, average daily time spent at home, regular exercise, smoking status, and alcohol consumption. Alcohol consumption was assessed using the following question: ‘During the past year, have you had any days on which you consumed 5 or more drinks in a day (for men) or 4 or more drinks in a day (for women)?’

#### 2.2.2. Home Injury Prevention Awareness

Home injury prevention awareness was assessed using an instrument developed by researchers based on a previous study [19,21,22]. The content validity and readability of the items were evaluated through a panel review involving five experts (one physician, two nursing professors, one gerontological nurse specialist, and one physical therapist). Item-level content validity indices (I-CVI) were calculated, and only items with a CVI of 0.80 or higher were retained in the final instrument. In addition, a pilot test was conducted with five older adults who met the inclusion criteria to assess clarity and comprehensibility. The questionnaire included the following items: perceived safety of the home environment (yes/no), interest in home injuries (yes/no), awareness of first aid for different injury mechanisms (yes/no), availability of an emergency kit (yes/no), participation in regular health check-ups (yes/no), frequency of home fire-safety inspections (yes/no), previous experience with injury-prevention education (yes/no), intention to participate in such education (yes/no), and perceived barriers to practicing preventive behaviors.

#### 2.2.3. Home Injury Prevention Behaviors

Home injury prevention behaviors were measured using a scale developed by researchers. Content validity and clarity were verified using the same expert review and pilot testing process as described above. The instrument consisted of 15 items, including statements such as “I use adequate lighting at night” and “I use handrails or safety bars in the bathroom.” Each item was rated on a 3-point scale (0 = never, 1 = sometimes, 2 = always), with higher total scores indicating greater engagement in injury-prevention behaviors. 

### 2.3. Data Collection 

This study was approved by the Institutional Review Board (CKU-25-02-0702). Participants were informed of the purpose and procedures of the study, the assurance of anonymity, and the confidentiality of their information before providing written informed consent. This study was conducted in accordance with the principles of the Declaration of Helsinki. Data collection was carried out from September to October 2025. The two principal researchers and one trained research assistant explained the study’s purpose to the administrators of each participating facility and obtained cooperation before conducting on-site data collection. Participants were recruited from older adults who were using the institution on the day of the researchers’ visit. On the survey day, the purpose and procedures of the study were explained, and questionnaires were distributed only to those who voluntarily agreed to participate and provided written informed consent. The researchers or the assistant read the questions aloud and recorded participants’ responses directly. Each survey took ~20 min to complete. All investigators and research assistants involved in data collection were external personnel with no employment, operational, or administrative affiliation with the participating institutions and received prior training on standardized survey procedures and ethical considerations to ensure that they did not influence participants’ responses during the survey process. Participants were provided with a small non-monetary token of appreciation (a household item) for their participation.

### 2.4. Data Analysis

Data were analyzed using the SPSS for Windows, version 27.0 (IBM Corp., Armonk, NY, USA). Descriptive statistics were used to summarize participants’ general characteristics and levels of awareness and behaviors related to home injury prevention. Differences in awareness and behaviors according to age and gender were analyzed using the chi-squared test, independent *t*-test, and one-way analysis of variance. 

## 3. Results

### 3.1. Participant Characteristics

Participants had a mean age of 77.8 years (range, 65–100), with the largest subgroup aged 75–84 years (n = 140, 46.8%). Women comprised 63.5% (n = 190). Most had an education level of middle school or below (n = 182, 60.9%) and had a spouse (n = 185, 61.9%); 69.9% were not employed (n = 209). Half reported exercising regularly (n = 150, 50.2%), 5.7% were current smokers (n = 17), and 14.1% reported alcohol consumption (n = 42). The most common daily time spent at home was approximately 13–20 h (n = 109, 36.5%) (Table 1). 

### 3.2. Differences in Home Injury Prevention Awareness According to Age and Gender

Interest in home injuries differed across age groups (*p* = 0.006); first-aid awareness differed for cuts/collisions (*p* = 0.002), choking/aspiration (*p* = 0.025), and dehydration/heat–cold illness (*p* < 0.001); possession of a home emergency kit (*p* = 0.003) and participation in regular health check-ups (*p* = 0.002) also differed by age. By gender, differences were observed in interest in home injuries (*p* < 0.001), first-aid awareness for fire, burns, and electrical accidents (*p* = 0.002), and intention to participate in prevention education (*p* = 0.007) (Table 2). 

### 3.3. Differences in Home Injury Prevention Behaviors According to Age and Gender

Overall levels of home injury-prevention behavior were higher among women than among men (*p* = 0.006). The most frequently cited barrier to practicing preventive or safety behaviors was “I often forget what I learned,” which predominated across all age groups and among women. Among men, the most common barrier was “lack of information” (Table 3). 

## 4. Discussion

This study explored older adults’ awareness and behaviors related to home injury prevention according to age and gender, ultimately to inform future directions for preventive education and safety-promotion interventions. When examining age-related differences in injury prevention awareness, the findings revealed that interest in home injury prevention declined with increasing age. This pattern may reflect a shift in perception among the oldest participants—viewing injuries as unavoidable consequences of aging rather than preventable events [23]. Previous studies similarly reported that older adults tend to exhibit lower awareness of injury prevention [9] and are less likely than younger individuals to perceive themselves as capable of improving their own safety [24]. Furthermore, evidence suggests that older adults have limited understanding of evidence-based preventive strategies compared with younger populations [25]. Declining physical health, frailty, and restricted mobility associated with advanced age [26,27] may also reduce both engagement in and perceived relevance of preventive behaviors. Nonetheless, given the higher incidence of falls and home injuries and the associated mortality among adults aged 80 years and older [17], interventions that raise risk awareness and motivate vigilance among the oldest old are urgently needed. Accordingly, strategies that incorporate educational programs tailored to the cognitive level of older adults are essential to promote interest in injury prevention and enhance sustained attention to safety.

As an indicator of injury prevention awareness, possession of an emergency kit at home was assessed in this study. Accordingly, in this study, 56.9% of participants reported keeping an emergency kit at home—slightly higher than the proportion reported among U.S. older adults (54.8%) [28] and substantially higher than that of older adults in China (17.79%) [29]. However, the rate of emergency kit possession was lowest among those aged 85 years and older (39.2%), mirroring their lower level of interest in injury prevention. Although several countries have promoted household emergency kit distribution as a policy initiative, mere availability of kits does not ensure preparedness. Without accompanying education, awareness, and motivation, proper maintenance and utilization remain limited [30]. Thus, programs should not only distribute emergency kits but also emphasize appropriate use, management, and upkeep, which are essential for enhancing sensitivity to emergencies and reduce complications through timely responses.

As another indicator of injury prevention awareness, regular health check-up participation was assessed. We also found that the rate of regular health check-ups decreased with age. This aligns with earlier reports that health-screening participation declines with increasing age [31] and that healthcare professionals tend to be less proactive in recommending screenings to those aged >80 years [32]. Although comprehensive full-body check-ups may not be necessary for every older adult, periodic assessments of sensory, musculoskeletal, and cognitive functions are critical for safety and injury prevention [14]. For older adults who already visit healthcare facilities regularly for chronic disease management, establishing an integrated care model that incorporates assessment of injury risk and preventive management within routine visits would be beneficial, for example, by linking brief risk screening during outpatient visits with tailored safety education and follow-up.

When examining gender-related differences in injury prevention awareness, female participants demonstrated a higher level of interest in home injury prevention than their male counterparts. This may be attributed to gender roles, as women typically assume domestic and caregiving responsibilities [33] that expose them to household hazards more frequently [18,20]. Women are also more likely to discuss health issues with others [34] and to be indirectly exposed to others’ injury experiences, factors that can heighten their awareness of prevention. Since perceived preventability strongly predicts engagement in protective behaviors [9], safety strategies targeting men—who demonstrated lower awareness—should emphasize the personal relevance and preventability of home injuries. In addition, approaches that reframe prevention messages, emphasize the tangible benefits of prevention, and incorporate social support [35] may be promising strategies for increasing men’s participation in injury prevention programs. 

Interestingly, male participants showed greater awareness of first-aid procedures for fire, burn, and electrical injuries than female participants. Previous research indicates that prior training and direct experience increase awareness and confidence in managing such emergencies [36]. Men may also be more likely to seek or be offered safety training opportunities related to fire or electrical hazards due to social expectations about their role in emergency response. Their higher exposure to manual labor and risky environments may further explain this pattern [37]. Additionally, although the differences did not reach statistical significance, awareness of first-aid procedures related to cuts/collisions and dehydration or heat- or cold-related illnesses tended to differ by gender, with older men showing greater awareness of first-aid procedures than older women. These findings suggested differences that may be attributable to gender-related variations in lifestyle patterns and experiences of risk exposure, indicating the need for more rigorous examination in future studies.

In this study, a greater proportion of older women expressed willingness to participate in preventive education compared with older men. Previous research has consistently shown that older women are more likely than men to seek safety and prevention services related to physical activity, fall prevention, and medication management, and to engage proactively with healthcare providers in discussions about injury prevention [38], which support the findings of this study. Moreover, recognition of the positive outcomes associated with fall-prevention activities enhances older adults’ intention to participate in such programs [39]. Notably, older men in this study demonstrated lower levels of interest in preventive activities and intention to attend educational programs. Educational interventions targeting older men should therefore emphasize that home injuries constitute tangible and personally relevant risks. Facilitating this shift in perception may foster motivation and lead to greater engagement in preventive behaviors. In addition, educational strategies that utilize male-friendly environments or group settings and incorporate elements of social support are likely to enhance participation in injury prevention programs among older men [40].

In the analysis of age- and gender-related differences in injury prevention behaviors, no significant differences were observed according to age. However, female participants demonstrated higher levels of preventive behavior than male participants, consistent with previous research [40]. Older women have been reported to show greater awareness of the need for safety equipment and installations [6]. This gender difference may be partly explained by women’s greater involvement in household activities related to home safety. Future studies should further examine how cultural and social factors influence gender differences in safety behavior among older adults.

Finally, when examining reasons for difficulty in performing injury prevention or safety behaviors, a tendency toward difficulty in maintaining preventive behaviors was observed across all age groups, as well as among women, with “I often forget what I learned” being the most commonly cited reason. Similarly, prior studies have shown that 57% of older adults adhered to at least one injury-prevention recommendation, while 32% could not recall any of the guidelines they had received [41]. Cognitive decline and memory impairment associated with aging may hinder older adults from retaining and applying safety knowledge over time [14]. Therefore, one-time educational interventions are unlikely to achieve sustained behavior change. Ongoing, tailored education that accommodates cognitive and health-literacy levels are crucial [42], and reminder systems or digital alerts could be explored [41]. Meanwhile, male participants most frequently cited “lack of information” as the main reason for difficulty engaging in preventive behaviors. This suggests that men may have fewer opportunities to access safety information or participate in educational programs. However, given the higher fatality rate from falls among older men [2], it is essential to design outreach programs that deliver safety information through venues and formats suited to their routines and interests, such as community centers or workplace-style group sessions. Furthermore, understanding why men report limited information access but low educational motivation is necessary; the content and delivery style of existing programs may not align with their learning preferences.

## 5. Limitation

This study has some limitations. The participants were community-dwelling older adults recruited from specific regions of Korea, which may restrict the generalizability of the findings to populations with differing cultural or environmental contexts. Since only individuals with intact cognitive function and independent communication abilities were included, the sample may not fully represent the broader diversity of the older population. In addition, cognitive function was not assessed using a standardized objective screening tool, and the potential impact of medication-related cognitive impairment, including effects associated with polypharmacy, was not fully considered. Therefore, future studies are recommended to incorporate objective cognitive assessments and to examine factors related to cognitive changes in older adults when analyzing study outcomes. The cross-sectional design also precludes causal inference regarding the relationship between awareness and preventive behaviors. Additionally, the use of self-reported questionnaires introduces the potential for response bias and social desirability effects. Although participants were asked about their awareness of first-aid procedures, their actual knowledge and skills were not objectively verified. Despite these constraints, the study offers meaningful insight into age- and gender-specific differences in awareness and behaviors related to home injury prevention, providing valuable evidence to guide the development of tailored educational and policy interventions for older adults.

## 6. Conclusions

These findings highlight the need for approaches that account for age- and gender-specific differences in both awareness and behavior when developing public health policies and preventive education programs to reduce home injuries among older adults. Educational and policy initiatives tailored to these characteristics may enhance safety, reduce the physical and social burden of household injuries, and ultimately improve overall quality of life in aging populations. 

## Figures and Tables

**Table 1:** Sociodemographic characteristics of the participants (n = 299).

Characteristics	Categories	n (%) or M ± SD
Age (years)		77.8 ± 7.0 (65–100)
	65–74	108 (36.1)
	75–84	140 (46.8)
	≥85	51 (17.1)
Sex	Male	109 (36.5)
	Female	190 (63.5)
Education	Middle School	182 (60.9)
	High School	80 (26.8)
	University	37 (12.4)
Marital status	without spouse	114 (38.1)
	with spouse	185 (61.9)
Employment	Yes	90 (30.1)
	No	209 (69.9)
Average time spent at home per day	<8 h	56 (18.7)
	8–12 h	95 (31.8)
	13–20 h	109 (36.5)
	>20 h	39 (13.0)
Exercise	Regular	150 (50.2)
	Occasionally	90 (30.1)
	Never	59 (19.7)
Smoking	Yes	17 (5.7)
	No	282 (94.3)
Alcohol consumption	Yes	42 (14.1)
	No	257 (85.9)

**Table 2:** Age- and gender differences in injury prevention awareness.

Characteristics	Total	Age			x^2^ (*p*)	Gender		x^2^ (*p*)
		65-74 (n = 108)	75-84 (n = 140)	≥85 (n = 51)		Male (n = 109)	Female (n = 190)	
	n (%)	n (%)	n (%)	n (%)		n (%)	n (%)	
Perception of home environment safety								
Safe	243 (81.3)	94 (87.0)	112 (80.0)	37 (72.5)	5.06 (0.080)	93 (85.3)	150 (79.0)	1.85 (0.174)
Unsafe	56 (18.7)	14 (13.0)	28 (20.0)	14 (27.5)		16 (14.7)	40 (21.0)	
Interest in home injury								
Yes	192 (64.2)	50 (46.3)	46 (32.9)	11 (21.6)	10.20 (0.006)	49 (44.9)	143 (75.3)	27.69 (<0.001)
No	107 (35.8)	58 (53.7)	94 (67.1)	40 (78.4)		60 (55.1)	47 (24.7)	
Awareness of First Aid for Emergencies *								
Falls	159 (53.2)	60 (55.6)	71 (50.7)	28 (54.9)	0.65 (0.724)	61 (56.0)	98 (51.6)	0.53 (0.465)
Cuts/Collisions	185 (61.9)	80 (74.1)	81 (57.9)	24 (47.1)	12.5 (0.002)	75 (68.8)	110 (57.9)	3.50 (0.062)
Fire/Burns/Electric shock	180 (60.2)	71 (65.7)	85 (60.7)	24 (47.1)	5.08 (0.079)	78 (71.6)	102 (53.7)	9.24 (0.002)
Choking/Swallowing difficulty	68 (22.7)	34 (31.5)	25 (17.9)	9 (17.7)	7.35 (0.025)	29 (26.6)	39 (20.5)	1.46 (0.227)
Drug-related accident	53 (17.7)	23 (21.3)	23 (16.4)	7 (13.7)	1.67 (0.435)	25 (22.9)	28 (14.7)	3.19 (0.074)
Dehydration/Heat or Cold-related illness	140 (46.8)	64 (59.3)	62 (44.3)	14 (27.5)	14.86 (<0.001)	59 (54.1)	81 (42.6)	3.68 (0.055)
Emergency Kit at home								
Yes	170 (56.9)	73 (67.6)	77 (55.0)	22 (39.2)	11.74 (0.003)	56 (51.4)	114 (60.0)	2.10 (0.147)
No	129 (43.1)	35 (32.4)	63 (45.0)	31 (60.8)		53 (48.6)	76 (40.0)	
Regular Health Check-ups								
Yes	267 (89.3)	103 (95.4)	125 (89.3)	39 (76.5)	12.95 (0.002)	98 (89.9)	169 (88.9)	0.07 (0.796)
No	32 (10.7)	5 (4.6)	15 (10.7)	12 (23.5)		11 (10.1)	21 (11.1)	
Fire Safety Inspection (gas, fire extinguisher, alarm system)								
Yes	200 (66.9)	78 (72.2)	89 (63.6)	33 (64.7)	2.19 (0.334)	69 (63.3)	131 (68.9)	0.99 (0.318)
No	99 (33.1)	30 (27.8)	51 (36.4)	18 (35.2)		40 (36.7)	59 (31.1)	
Experience of Preventive Education								
Yes	96 (32.1)	34 (31.5)	45 (32.1)	34 (66.7)	0.05 (0.973)	32 (29.4)	64 (33.7)	0.59 (0.441)
No	203 (67.9)	74 (68.5)	95 (67.9)	17 (33.3)		77 (70.6)	126 (66.3)	
Intention to Participate in Prevention Education								
Yes	241 (80.6)	85 (78.7)	116 (82.9)	40 (78.4)	1.58 (0.813)	81 (74.3)	160 (84.2)	9.56 (0.007)
No	37 (12.4)	15 (13.9)	14 (10.0)	8 (15.7)		22 (20.2)	15 (7.9)	
Not sure	21 (7.0)	8 (7.4)	10 (7.1)	3 (5.9)		6 (5.5)	15 (7.9)	

* Percentage of respondents aware of first-aid methods by injury mechanism.

**Table 3:** Age- and gender differences in injury prevention behaviors.

Characteristics	Total	Age			F (*p*)	Gender		t (*p*)
		65-74 (n = 108)	75-84 (n = 140)	≥85 (n = 51)		Male (n = 109)	Female (n = 190)	
	N (%) or M ± SD	N (%) or M ± SD				N (%) or M ± SD		
Injury prevention behaviors	2.39 ± 0.35	2.43 ± 0.31	2.36 ± 0.38	2.41 ± 0.32	0.43 (0.651)	2.312 ± 0.38	2.44 ± 0.32	−2.78 (0.006)
Reasons for Difficulty in Performing Injury Prevention or Safety Behaviors **								
Forget what was learned	98 (32.8)	30 (27.8)	54 (38.6)	14 (27.5)		29 (26.6)	69 (36.3)	
Do not feel the need	69 (23.1)	28 (25.9)	31 (22.1)	10 (19.6)		28 (25.7)	41 (21.6)	
Lack of information	66 (22.1)	26 (24.1)	34 (24.3)	6 (11.8)		35 (32.1)	31 (16.3)	
No one to assist	63 (21.1)	15 (13.9)	34 (24.3)	14 (27.5)		24 (22.0)	39 (20.5)	
Physical discomfort	48 (16.1)	10 (9.3)	23 (16.4)	15 (29.4)		12 (11.0)	36 (19.0)	
Difficulty modifying home environment	22 (7.4)	7 (6.5)	11 (7.9)	4 (7.8)		10 (9.2)	12 (6.3)	
Financial burden	16 (5.4)	5 (4.6)	9 (6.4)	2 (3.9)		10 (9.2)	6 (3.2)	

** multiple choice.

## Data Availability

The data are not publicly available due to ethical and privacy considerations.
